# A simple combined antegrade and retrograde dilatation technique

**DOI:** 10.4103/0971-9261.43824

**Published:** 2008

**Authors:** Subhasis Roy Choudhury, Pinaki R. Debnath, Anand S. Kushwaha, Rajiv Chadha

**Affiliations:** Department of Pediatric Surgery, Lady Hardinge Medical College and Associated Kalawati Saran Children’s Hospital, New Delhi - 110 001, India

**Figure 1:**
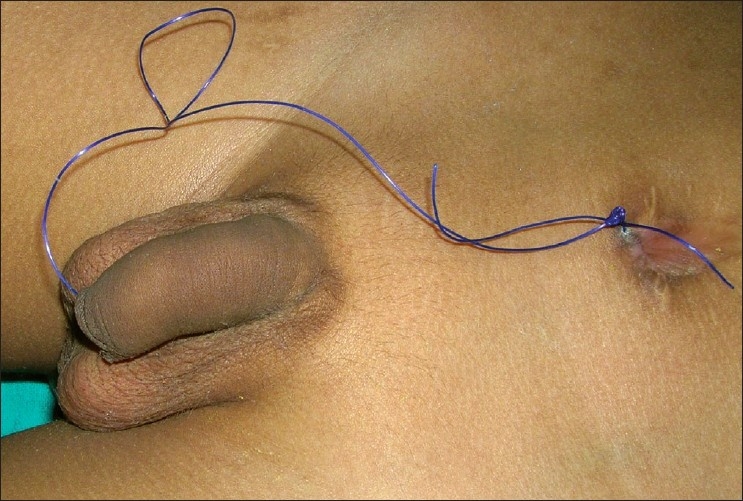
Urethral trans-anastomotic thread following end-to-end anastomosis

Sir,

Anastomotic stricture following complex esophageal and urethral anastomosis is often a distressing problem. Transanastomotic stenting and postoperative dilatation appears to be a useful adjunct in the management of this problem. Combined antegrade and retrograde dilatation is a very useful technique for difficult strictures.[1–3] We have used a simple novel technique of combined antegrade and retrograde dilatation after difficult esophageal and urethral anastomosis with satisfactory result. Eight cases, four each of difficult esophageal and urethral end-to-end anastomosis were dilated using this technique. Three cases had long-gap esophageal atresia. Initial esophagostomy and subsequent esophageal lengthening followed by an end-to-end esophageal anastomosis was performed. Since a high incidence of esophageal anastomotic strictures were reported following this procedure,[4–5] we stented the anastomosis with a Ryles tube of appropriate size and prophylactically started dilating the esophagus after two weeks. The trans-anastomotic stent was replaced with a trans-anastomotic thread of thick nylon or silk, and the ends were secured outside the nose and abdominal wall at the gastrostomy site. The dilatation was performed under a short general anesthesia by tying a Ryles tube or a Foley catheter of appropriate size to the trans-anastomotic thread at the gastrostomy site and pulling out the other end of the thread through the mouth or nose. Similar dilatation technique was applied in another patient after resection and end-to-end anastomosis of a corrosive stricture of middle third of the esophagus. The interval and number of dilatations varied, however, an average of three dilatations at an interval of two weeks was found to be effective. The trans-anastomotic thread was left for a long time for any future need of dilatation. No complication following the dilatation was encountered. Four cases of traumatic stricture of the membranous urethra underwent end-to-end anastomosis over a trans-anastomotic stent (a silicon Foley catheter), which was tied to a suprapubic catheter (Malecot). Three weeks later the stent was replaced with a thick prolene thread, the two ends of which were secured at the external meatus and the suprapubic site [Figure 1]. Combined antegrade and retrograde dilatations were performed by tying a Ryles tube or a Foley catheter of appropriate size to the thread. An average of three dilatations at two weeks interval was found to be sufficient. No complication was encountered. Various techniques are available for dilatation of the esophagus and urethra; however, these techniques require the availability of endoscopy, fluoroscopy, various dilators, guide wire and balloon. Our technique of simple combined antegrade and retrograde dilation of the esophagus and urethra is unique. The merits of the technique are as follows: 1) simple; 2) quick; 3) safe, as chance of perforfation or false passage is remote; 4) effective; and 5) does not require fluoroscopy or any special dilator.
